# The ARSACS disease protein sacsin controls lysosomal positioning and reformation by regulating microtubule dynamics

**DOI:** 10.1016/j.jbc.2022.102320

**Published:** 2022-08-04

**Authors:** Vincent Francis, Walaa Alshafie, Rahul Kumar, Martine Girard, Bernard Brais, Peter S. McPherson

**Affiliations:** Department of Neurology and Neurosurgery, Montreal Neurological Institute, McGill University, Montreal, Quebec, Canada

**Keywords:** ataxia, microtubules, lysosomes, autophagy, neurodegeneration, ALR, autolysosome reformation, ARSACS, autosomal recessive spastic ataxia of Charlevoix-Saguenay, KD, knockdown, MAP, MT-associated protein, MT, microtubule, PLL, poly-L-lysine

## Abstract

Autosomal recessive spastic ataxia of Charlevoix-Saguenay is a fatal brain disorder featuring cerebellar neurodegeneration leading to spasticity and ataxia. This disease is caused by mutations in the *SACS* gene that encodes sacsin, a massive 4579-amino acid protein with multiple modular domains. However, molecular details of the function of sacsin are not clear. Here, using live cell imaging and biochemistry, we demonstrate that sacsin binds to microtubules and regulates microtubule dynamics. Loss of sacsin function in various cell types, including knockdown and KO primary neurons and patient fibroblasts, leads to alterations in lysosomal transport, positioning, function, and reformation following autophagy. Each of these phenotypic changes is consistent with altered microtubule dynamics. We further show the effects of sacsin are mediated at least in part through interactions with JIP3, an adapter for microtubule motors. These data reveal a new function for sacsin that explains its previously reported roles and phenotypes.

ARSACS (autosomal recessive spastic ataxia of Charlevoix-Saguenay) is a progressive neurodegenerative disorder characterized by loss of cerebellar Purkinje neurons. Initially identified in the Charlevoix and Saguenay regions of Quebec (1, 2), the disease is now recognized worldwide and is the second most common recessive form of ataxia (3). Clinical features vary depending on the patient population but in the Quebec population, ARSACS features spasticity, ataxia, polyneuropathy, and retinal thickening (1). Patients display an unsteady gait, become wheelchair bound at an average age of 41, and have a reduced life expectancy. The *SACS* gene, mutations in which are responsible for ARSACS, encodes a massive 4579 amino acid (521 kDa) protein (2). Since the initial discovery of the founder mutation (c.8844 delT) in French-Canadian ARSACS patients, approximately 200 mutations have been identified spanning the full length of the protein (4).

Sacsin is a multi-modular protein consisting of an ubiquitin-like domain, which binds to the proteasome (5), three large sacsin repeat regions suggested to have Hsp90-like chaperone function (6), a potential XPCB domain that binds the Ube3A ubiquitin protein ligase (7), a DnaJ domain that interacts with Hsc70 (5), and a HEPN domain mediating sacsin dimerization (8). This domain organization suggests a functional role for the protein in proteostasis. However, the cellular function of sacsin remains largely unknown and there are no therapies available for ARSACS.

We previously demonstrated that in primary neurons and nonneuronal cell lines, depletion of sacsin with inhibitory RNA results in a hyperfused mitochondrial network (9). Moreover, mitochondria accumulate in neuronal cell bodies suggesting a mitochondrial transport defect that could result from the altered mitochondrial network or from a primary defect in organelle transport (9). A subsequent study reported decreased mitochondrial motility in the axons of *Sacs−/−* motor neurons, arguing for a primary transport defect (10). This study also reported an accumulation of abnormal, nonphosphorylated neurofilaments in the somatodendritic region of various neuronal population in *Sacs*−/− mice (10). In fibroblasts from ARSACS patients, intermediate filaments are present as collapsed bundles close to the microtubule (MT) organizing center (11). These cellular phenotypes are consistent with a functional role for sacsin in regulating cytoskeleton dynamics.

To further characterize the physiological function of sacsin and to test the hypothesis that sacsin regulates organelle positioning by regulating cytoskeletal dynamics, we used sacsin KO cells, primary neuronal cultures from *sacs* KO mice, and ARSACS patient fibroblasts. Our results reveal that lysosome positioning and motility are disrupted upon loss of sacsin. We demonstrate that sacsin binds to MTs and regulates MT dynamics. We also identify a novel interaction of sacsin with JIP3, an adapter between MTs and organelles, and we provide evidence that this interaction is crucial in controlling lysosome positioning. The regulation of MT dynamics provides a unifying hypothesis that can explain all known sacsin loss-of-function phenotypes.

## Results

### Sacsin regulates lysosome positioning

**Figure 1:**
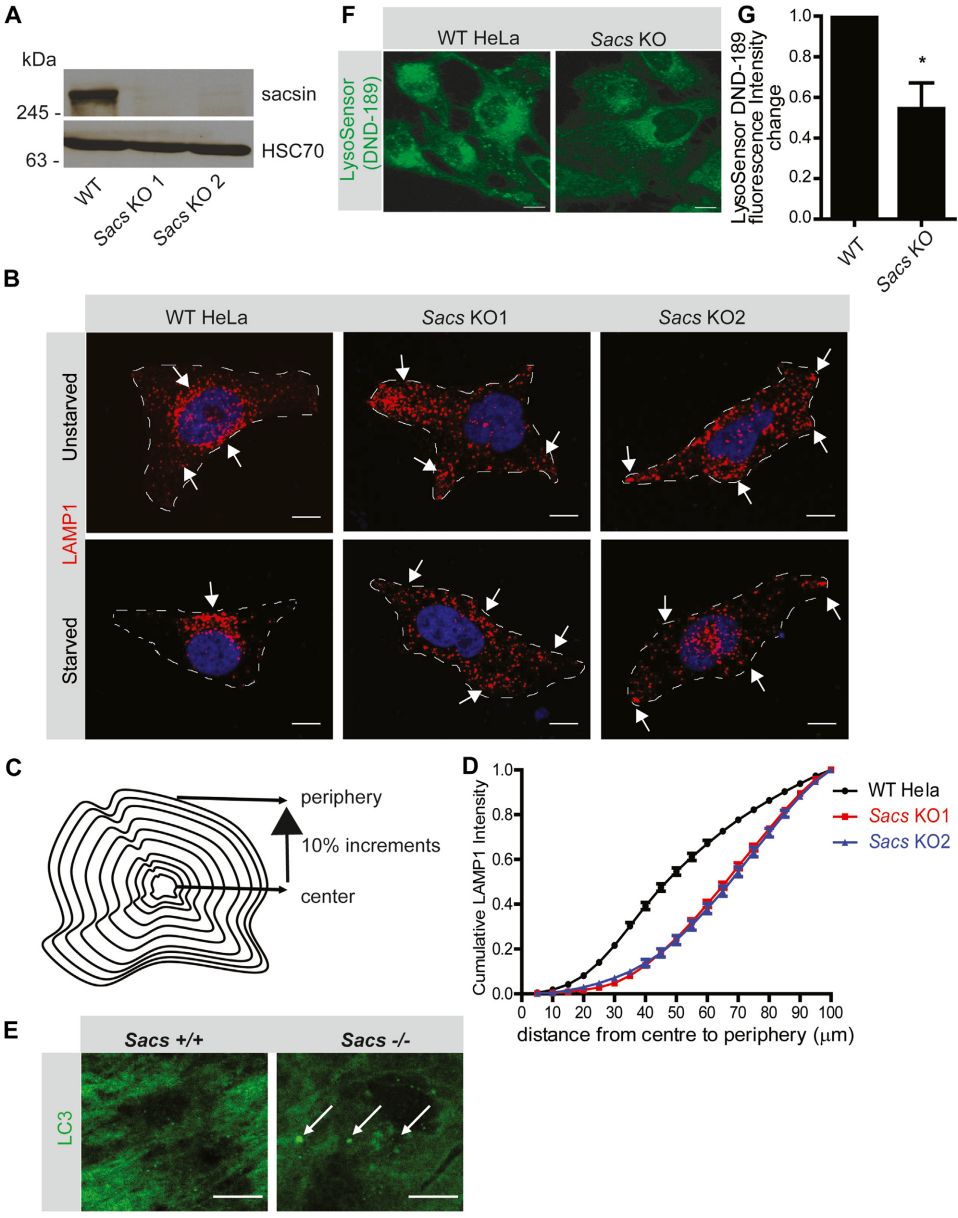
**Sacsin regulates lysosomal positioning.***A*, lysates were prepared from WT and sacsin KO HeLa cells and processed for immunoblot with antibodies recognizing sacsin and HSC70. *B*, representative confocal immunofluorescence images revealing LAMP1 distribution in WT and sacsin KO HeLa cells under nutrient-rich conditions (unstarved) and starvation conditions. DAPI was used to stain nuclei. The scale bar represents 10 μm. *C*, schematic showing the application of LAMP1 distribution in HeLa cells. *D*, cumulative LAMP1 distribution intensity plot of sacsin KO and WT cells under conditions of starvation. Data represents n = 40 cells. *E*, cerebellar sections of WT and sacsin KO mice were stained for LC3. The scale bar represents 20 μm. *F*, LysoSensor DND-189 fluorescence in WT and sacsin KO cells. The scale bar represents 5 μm. *G*, graph showing fluorescence intensity change of LysoSensor DND-189 in WT HeLa and sacsin KO cells. Data represented as mean ± SEM. ∗*p* < 0.05.

**Supplemental Figure S1:**
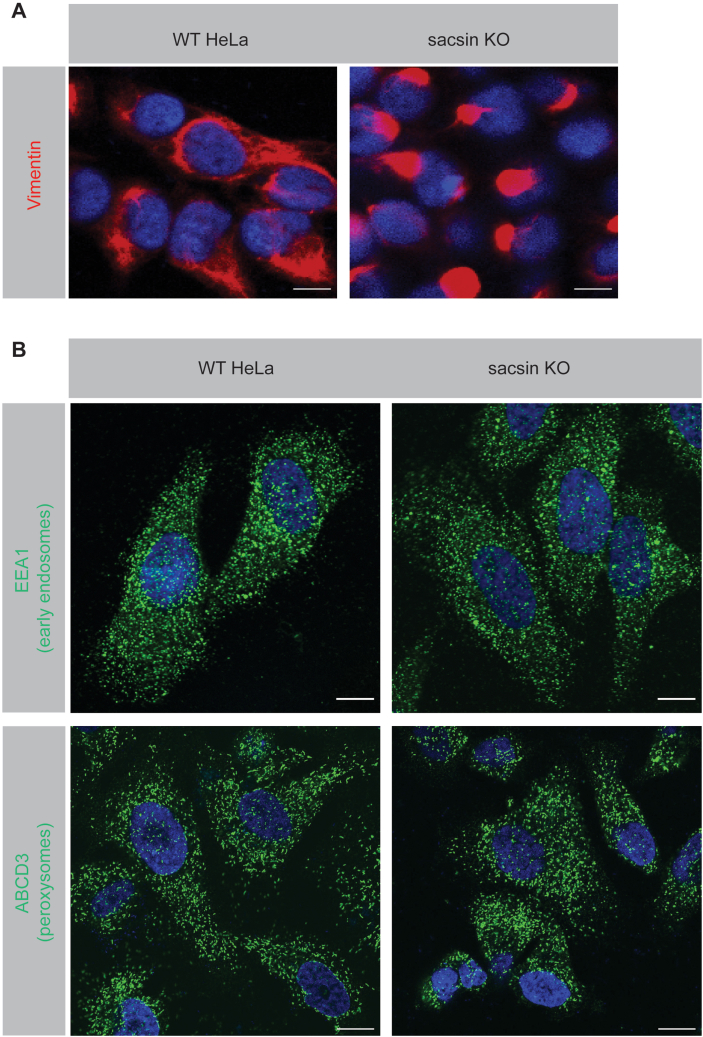
**Distribution of mitochondria and early endosomes in control and ARSACS knockout cells.***A*, HeLa cells, WT, or sacsin KO were stained with vimentin. *B*, HeLa cells, WT, or sacsin KO were stained with antibodies to detect early endosomes (EEA1) and peroxisomes (ABCD3) respectively. The scale bars represent 10 μm.

**Supplemental Figure S2:**
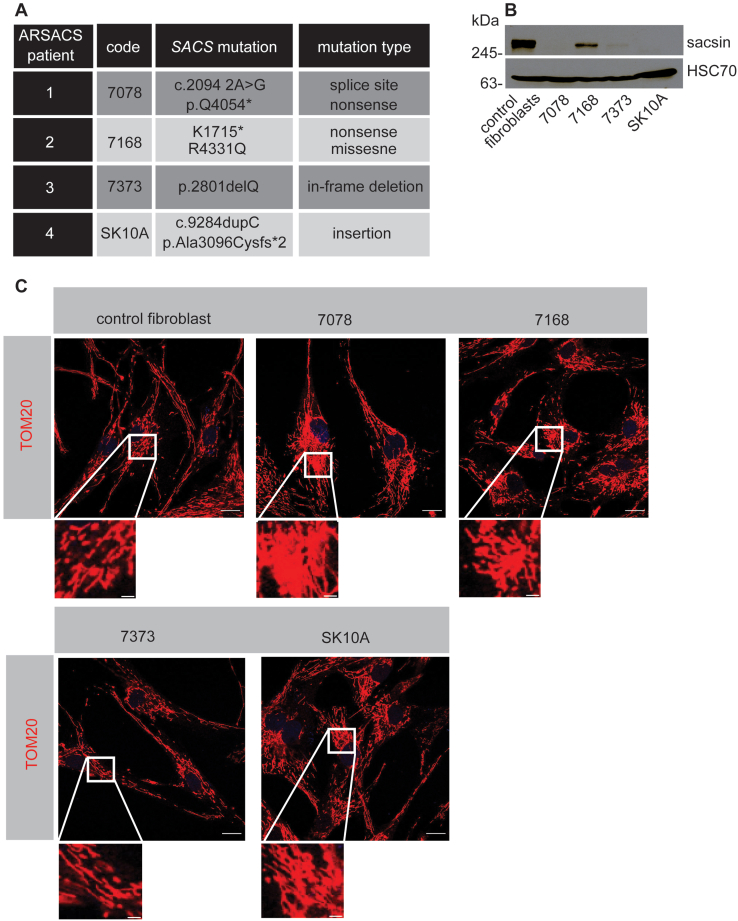
**ARSACS-derived patient fibroblast cells.***A*, table describing ARSACS patient fibroblasts. *B*, immunoblot showing sacsin expression in various ARSACS patient cell lines. *C*, mitochondria staining with TOM20 in patient fibroblasts. *Boxed areas* denote higher magnification. The scale bar represents 10 μM and 2 μM for low- and high-magnification images.

Reduction of sacsin in neurons using inhibitory RNA results in the accumulation of mitochondria in cell bodies and decreased rates of mitochondrial transport in axons, suggesting an organelle transport function for the protein (9, 10). To further test for sacsin involvement in organelle transport or positioning, we generated sacsin KO HeLa cells using CRISPR/Cas9, leading to complete loss of sacsin protein in two independent lines (Fig. 1*A*). Similar to previous reports, sacsin KO cells display vimentin bundling (11) (Fig. S1*A*). We examined for changes in organelle positioning and discovered that the distribution of lysosomes is altered in sacsin KO cells compared to WT cells. Specifically, in KO cells under steady-state conditions, lysosomes stained with LAMP1 display a peripheral distribution with little juxtanuclear accumulation, whereas in WT cells, lysosomes have a more typical juxtanuclear pattern with seemingly less peripheral staining (unstarved, Fig. 1B). In contrast, there was no change in the distribution of early endosomes or peroxisomes (Fig. S1*B*). As expected, mitochondria were hyperfused in KO cells and fibroblasts from ARSACS patients (Fig. S2, *A*–*C*).

Lysosomes are highly dynamic organelles, sometimes stationary, but often moving between a perinuclear pool and a more distributed peripheral pool (12). The positioning of lysosomes controls their activity and contributions to cellular functions including responses to changing nutrient levels, autophagy, antigen presentation, cell adhesion, cell migration, and cancer cell invasion (13). For example, starvation induces autophagy and stimulates repositioning of lysosomes from the periphery to the perinuclear pool to facilitate autophagosome/lysosome fusion (14). We thus starved cells and as expected, in WT cells, starvation repositions lysosomes, which show an even more clustered juxtanuclear distribution than under steady-state conditions (Fig. 1*B*). In KO cells, lysosomes remain scattered with little evidence of juxtanuclear accumulation (Fig. 1*B*).

**Supplemental Figure S3:**
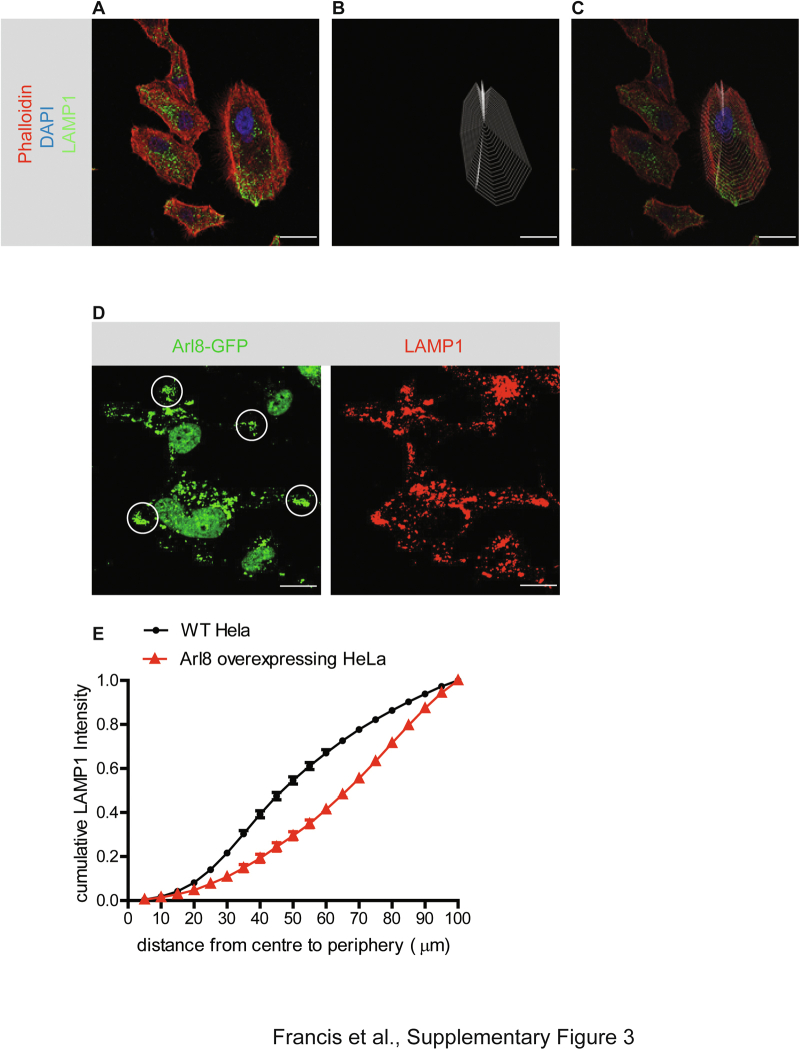
**Pipeline for quantification of lysosome distribution.***A*, composite image of HeLa cell with cell body stained with phalloidin (*red*), lysosomes (*green*), and nucleus (*blue*). *B*, contours with 10% increments in distance overlaid on the cell is considered. *C*, concentric contours from the cell center to the periphery with 10% increment in distance overlaid on the cell is considered. *D*, representative confocal image showing GFP-Arl8B overexpression in HeLa cells. *E*, Cumulative Lamp1 distribution intensity plot showing lysosome distribution upon GFP-Arl8 overexpression. The scale bars represent 5 μm.

To quantify alterations in lysosome positioning resulting from sacsin KO, we developed a lysosome distribution measure. Cells were outlined and concentric rings were drawn at 10% intervals based on the shape of the cell (Fig. 1*C*). The cumulative LAMP1 intensity was then plotted relative to the whole cell from cell center to periphery (Figs. 1*D* and S3, *A*–*C*). Shifting of the cumulative LAMP1 intensity curve toward the left demonstrates a perinuclear distribution whereas a shift toward the right indicates a peripheral distribution. Sacsin KO lines had a significant shift of lysosomal distribution to the periphery as revealed by rightward shifts in the distribution curves (Fig. 1*D*). To further validate our quantification assay, we manipulated lysosome distribution by overexpression of GFP-Arl8 (Fig. S3*D*), which is known to reposition lysosomes to the periphery (15) and plotted the LAMP1 distribution (Fig. S3*E*). Overexpression of GFP-Arl8 shifted the curve toward the right demonstrating a more peripheral distribution of lysosomes (Fig. S3*E*). The source code and the implementation of this algorithm are available as an open source code at GitHub (https://github.com/Vincent-Francis/Quantification-LysosomeDistribution). Since the position of lysosomes within cells helps determine their luminal pH, we sought to measure the acidity levels of lysosomes in WT and sacsin KO cells using LysoSensor DND-189. In acidic organelles, the fluorescence intensity of LysoSensor DND-189 depends on the acidity levels. We observed decreased levels of acidic lysosomes in sacsin KO cells compared to WT cells (Fig. 1, *F* and *G*). Since autophagic clearance of degradative cargo by lysosomes is dependent both on lysosome position and acidity, we performed immunofluorescence on WT and sacsin KO mice brains to see if there are any defects in autophagy using LC3, an autophagosome marker. We observed accumulation of LC3 in sacsin KO mice compared to WT controls (Fig. 1*E*).

**Figure 2:**
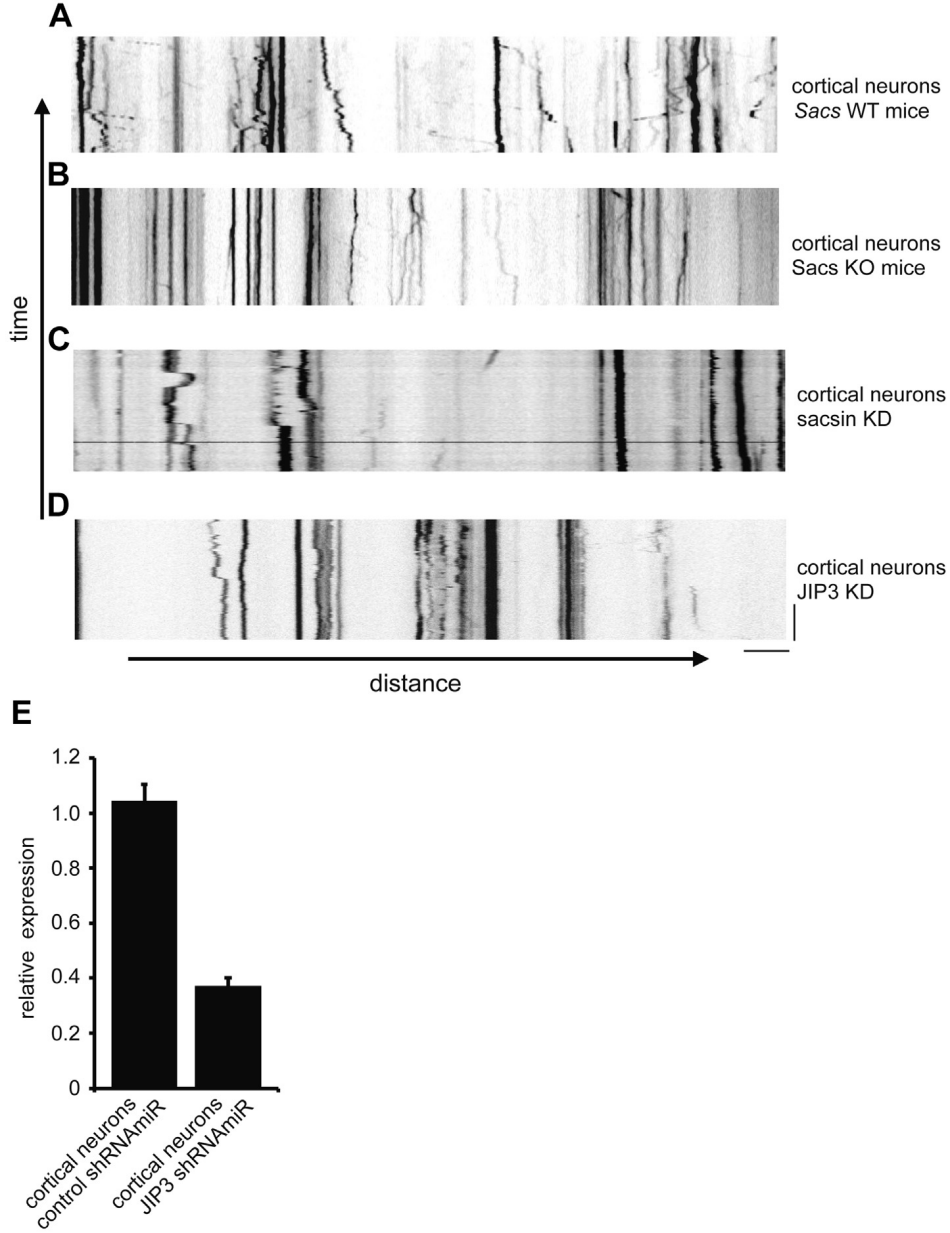
**Sacsin regulates lysosomal motility in neurons.***A*, cortical neurons from sacsin WT mice were incubated with lysotracker and imaged live with images captured every 1 s for 70 cycles. The kymograph represents lysosome transport. The movement of the lysotracker-labeled lysosomes on the vertical axis over time indicates the transport of lysosomes. *B*, kymograph representing lysosome trafficking in cortical neurons as in (*A*) but prepared from sacsin KO mice. *C*, kymograph representing lysosome trafficking in primary cortical neurons following sacsin knockdown (KD). *D*, kymograph representing lysosome trafficking in primary cortical neurons following JIP3 knockdown (KD). *E*, cortical neurons were transduced with lentivirus driving a control shRNAmiR or an shRNAmiR targeting JIP3. The scale bar represents 5 μm. qPCR was used to determine the relative levels of JIP3 mRNA. The bars represent mean ± SEM; n = 3.

**Supplemental Figure S4:**
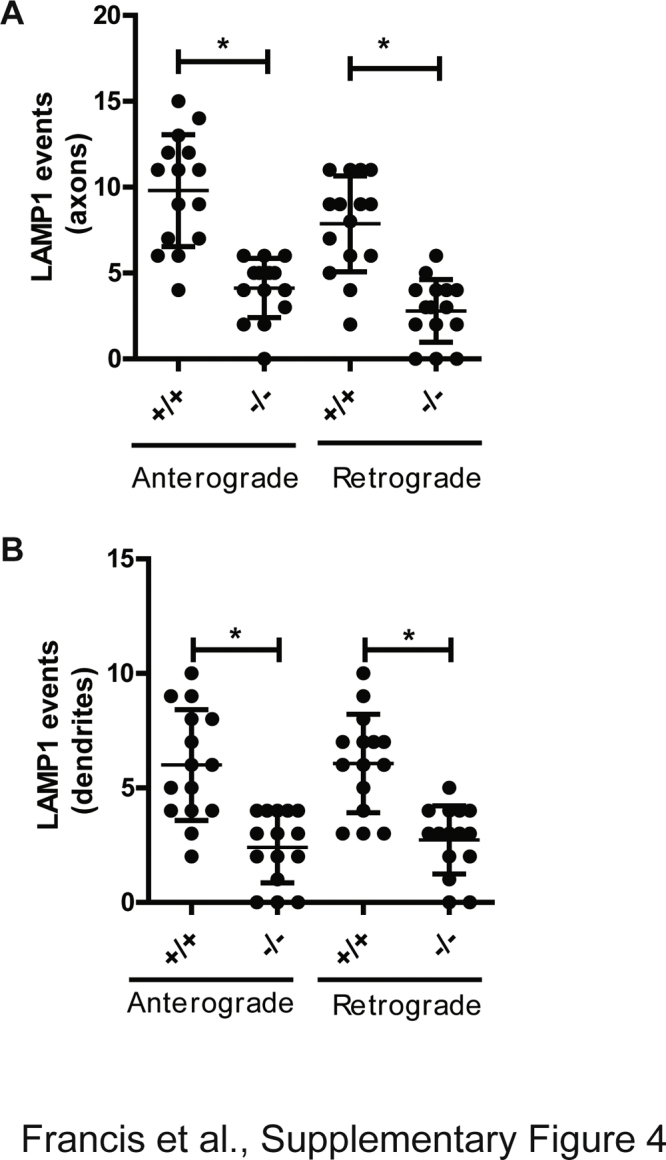
*A*, quantitative analysis of lysosome movement in axons from kymographs of WT neurons (n = 15) and sacsin KO neurons (n = 15). *B*, quantitative analysis of lysosome movement in dendrites from kymographs of WT neurons (n = 15) and sacsin KO neurons (n = 15). Statistical significance was calculated by one-way ANOVA followed by Dunnett’s test ∗*p* < 0.01.

Since ARSACS is primarily a neuronal disease, we tested to see if lysosome positioning or transport is affected in neurons following loss of sacsin. Sacsin KO mice are a robust model of the disease revealing phenotypes that reflect symptoms seen in human patients (10). We generated cultures of cortical neurons from WT and KO mice and stained lysosomes with lysotracker. In WT neurons, lysosomes are concentrated in cell bodies (arrows, Video S1) but are also detected throughout neuronal processes where many are motile. In sacsin KO neurons, the overall distribution of lysosomes between cell bodies and processes is similar to WT (arrows indicate multiple cell bodies in the image), but mobility is strongly reduced with a higher proportion of stationary lysosomes (Video S2). The altered motility in neuronal processes is most obvious when presented in the form of kymographs, revealing that many lysosomes are stationary in the sacsin KO neurons compared to WT neurons (Fig. 2, *A* and *B*). In addition, we used validated small inhibitory RNA sequences specific for rat sacsin, prepared in a miRNA backbone (shRNAmiR) and packaged into lentivirus to knockdown (KD) sacsin in cultured rat cortical neurons, as previously described (9). Reduced lysosomal mobility was also observed in sacsin KD neurons (Video S3 and Fig. 2*C*) as compared to control shRNAmiR-treated cells (Video S4). To further examine lysosome dynamics, we quantified the anterograde and retrograde trafficking of lysosomes in WT and sacsin KO neurons. To study this, DIV8 WT and sacsin KO neurons were labeled with lysotracker, and live cell imaging was performed. We observed that the number of lysosomes moving in both anterograde and retrograde directions were reduced in both axons and dendrites (Fig. S4, *A* and *B*). Together, these data indicate that sacsin plays a role in lysosomal trafficking and positioning in both neurons and nonneuronal cells.

### Sacsin interacts with JIP3 to regulate lysosomal positioning and function

**Figure 3:**
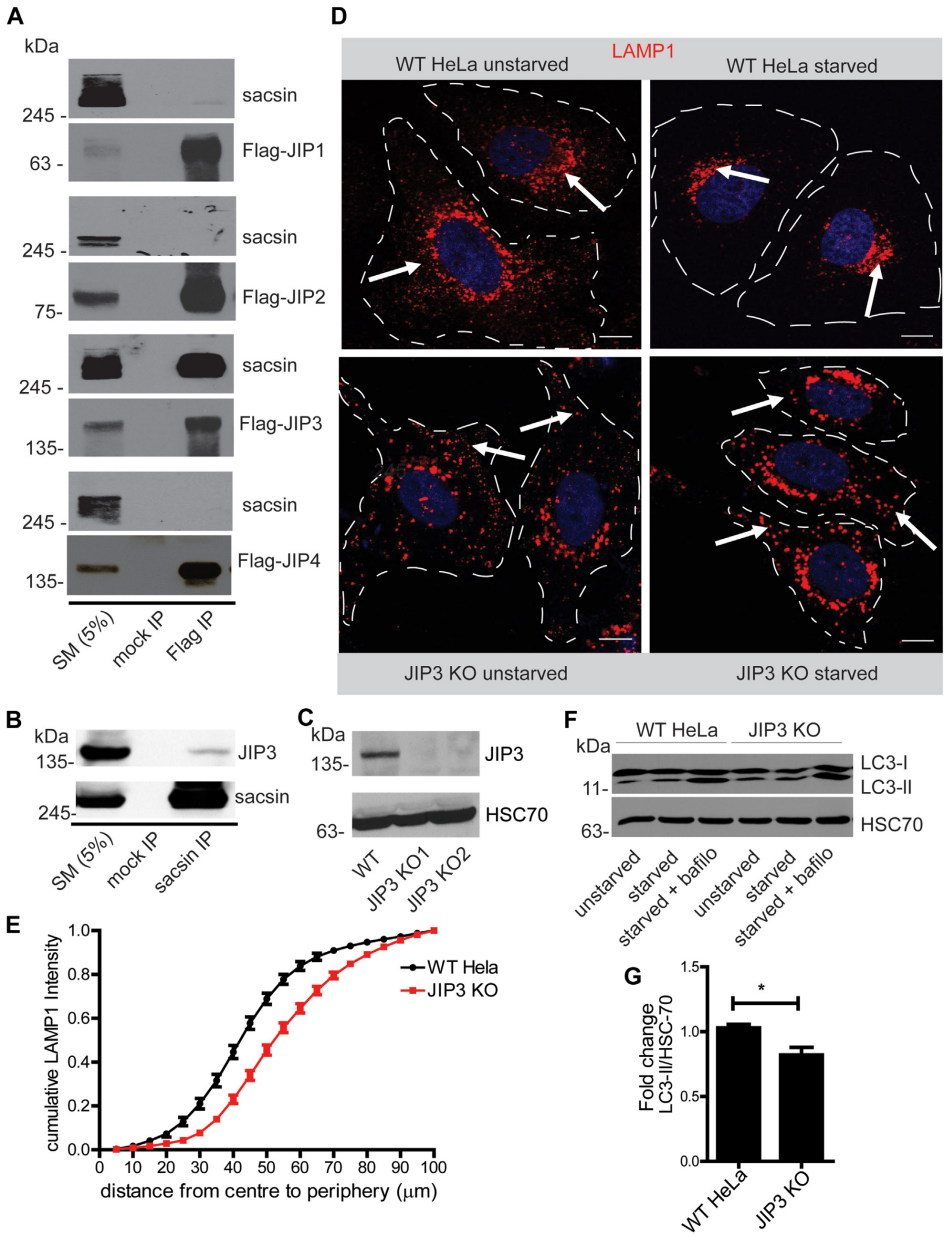
**Sacsin interacts with JIP3 to control lysosomal positioning.***A*, lysates prepared from HEK-293 cells transfected with Flag-JIP1, 2, 3, and 4 were processed for immunoprecipitation with antibody recognizing Flag. The precipitated samples were processed for immunoblot with antibodies recognizing Flag and sacsin as indicated. An aliquot of the cell lysates (starting material, SM) equal to 5% of that added to the immunoprecipitates were analyzed in parallel. The apparent molecular mass of sacsin and the Flag-tagged constructs are indicated. *B*, lysates prepared from mouse brain were processed for immunoprecipitation with antibody recognizing sacsin. The precipitated samples were processed for immunoblot with antibodies recognizing JIP3 and sacsin as indicated. *C*, lysates were prepared from WT and JIP3 KO HeLa cells and processed for immunoblot with antibodies recognizing JIP3 and HSC70. *D*, representative confocal images showing the distribution of LAMP1 in cells treated with control HeLa (*top*) or JIP3 KO (*bottom row*) under unstarved or starved conditions, as indicated. The scale bar represents 5 μm. *E*, cumulative LAMP1 distribution intensity plot of JIP3 KO and WT cells under conditions of starvation. *F*, immunoblot of lysates from WT HeLa and JIP3 KO cells which were starved in EBSS for 3 h or starved with 100 nM bafilomycin in EBSS for 3 h. *G*, graph showing quantification of LC3 II/HSC70 ratios from WT HeLa and JIP3 KO cells and represented as mean ± SEM using two tailed *t* test ∗*p* < 0.05.

To further elucidate the molecular mechanisms involved in sacsin regulation of lysosomal positioning and transport, we performed mass spectrometry analysis of sacsin immunoprecipitates and identified JIP3 (C-jun-amino-terminal kinase-interacting protein 3) as a sacsin-interacting partner. The mass spectrometry proteomics data have been deposited to the ProteomeXchange Consortium *via* the PRIDE partner repository with the dataset identifier PXD033823 (Supplementary Data File 1). This is particularly relevant as JIP3 functions as an adapter for MT-dependent organelle transport, including that of lysosomes (16). The sacsin–JIP3 interaction was further validated through immunoprecipitation experiments. JIP3 belongs to the JIP family of proteins that comprises four isoforms (JIP1–4) (17). The four JIP isoforms, each with an N-terminal Flag tag, were immunoprecipitated with anti-Flag antibody following transfection in HEK-293 cells. The immunoprecipitates were then immunoblotted with antibodies recognizing Flag and sacsin (Fig. 3*A*). Endogenous sacsin coimmunoprecipitates with JIP3 but not with the other three JIP isoforms (Fig. 3*A*). We also detect interaction of sacsin with endogenous JIP3 (Fig. 3*B*).

To further investigate the role of JIP3 in lysosome dynamics, we generated a JIP3 KO HeLa cell line using CRISPR/Cas9, leading to complete loss of protein in two independent KO lines (Fig. 3*C*). In WT cells, lysosomes are in both peripheral and juxtanuclear pools, and starvation causes a redistribution toward the juxtanuclear pool (Fig. 3*D*). In JIP3 KO cells, LAMP1-positive lysosomes have a more peripheral distribution and fail to redistribute toward the juxtanuclear area following nutrient deprivation (Fig. 3*D*), similar to what is seen in sacsin KO cells (Fig. 1). The cumulative LAMP1 intensity was plotted for WT and JIP3 KO cells under conditions of starvation and revealed a similar shift as for the sacsin KO cells (Figs. 1*D* and 3*E*).

Since lysosomal positioning is important for facilitating the fusion of autophagosomes with lysosomes, the failure of lysosomes to reposition in JIP3 KO cells could impair autophagy. To test if autophagy is altered in JIP3 KO HeLa cells, both WT and KO cells were starved and treated with bafilomycin and LC3 levels were determined. We found that autophagy was impaired in JIP3 KO cells, as reduced levels of LC3-II were observed in JIP3 KO cells when compared to control (Fig. 3, *F* and *G*).

We also tested the loss of JIP3 on lysosome dynamics in primary rat neuronal cultures. Neurons were transduced with a lentivirus driving JIP3-specific shRNAmiR and efficient KD was confirmed by qPCR (Fig. 2*E*). Compared to control shRNAmiR-treated neurons (Video S4), JIP3 KD neurons contain seemingly aggregated lysosomes in neuronal processes (Video S5). The control-treated neurons showed bidirectional lysosomal trafficking within neuronal processes, whereas JIP3 KD neurons displayed a significantly higher number of stationary lysosomes (Video S5 and Fig. 2*D*). These results are similar to those observed with sacsin KD and to an earlier study where disruption of JIP3 led to altered axonal transport of lysosomes and promoted amyloid plaque pathology (18).

### Sacsin regulates MT dynamics

**Figure 4:**
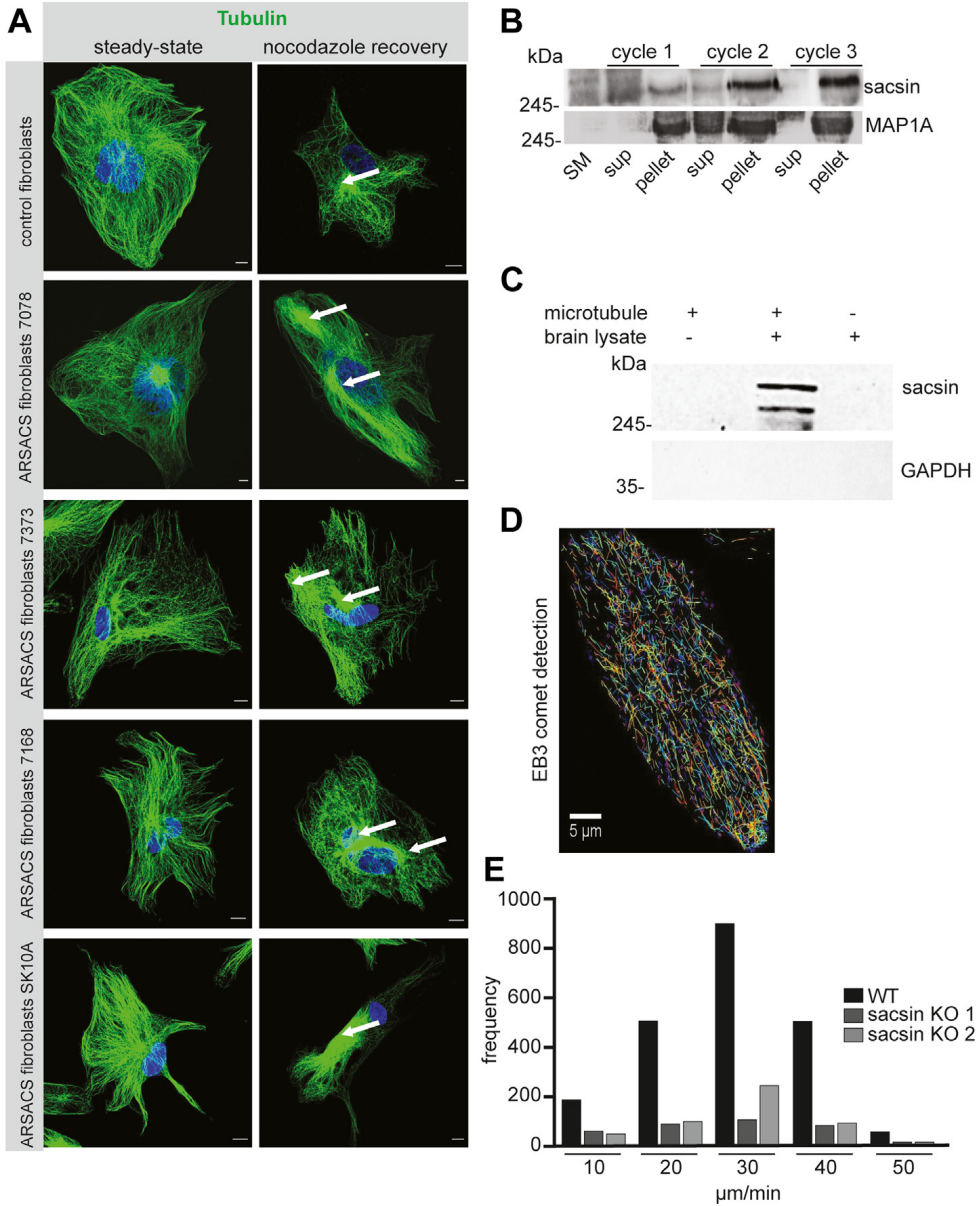
**Sacsin binds to MTs and regulates MT dynamics.***A*, control fibroblasts or fibroblasts from various ARSACS patients were stained to reveal MT networks either at steady-state or following 1 h of recovery after complete depolymerization of MT networks with nocodazole. *Arrows* show bundled formation of MT in ARSACS fibroblasts. The scale bars represent 10 μm. *B*, brain extracts were prepared and passed through a protocol involving cycles of polymerization and depolymerization of MTs with related ultracentrifugation steps to generate supernatant (sup) and pellet fractions. Each fraction, along with an aliquot of the original brain lysate (starting material, SM), were prepared for immunoblot with antibodies recognizing the indicated proteins. The apparent molecular mass of the proteins is indicated. *C*, purified MTs, stabilized with taxol, were incubated with solubilized brain lysates prepared from WT or sacsin KO mice. The samples were processed by ultracentrifugation and the pellets were prepared for immunoblot with antibodies recognizing the indicated proteins. *D*, representative image showing EB3 tracking in WT HeLa cells which were transiently transfected with EB3-GFP. The scale bar represents 5 μm. *E*, plot showing MT growth rates in WT and sacsin KO cells, which were transiently transfected with EBP3-GFP, as described in (*D*). More than 100 MT plus ends were tracked for each condition. ARSACS, autosomal recessive spastic ataxia of Charlevoix-Saguenay; MT, microtubule.

Organelle transport is mediated primarily by MTs and their associated adapters (19). We hypothesized that mispositioning of lysosomes upon loss of sacsin and JIP3 could be due to alterations in MT-based transport and/or MT dynamics. We thus investigated MT growth dynamics in WT and ARSACS fibroblasts. MTs were depolymerized using nocodazole and then allowed to regrow after drug washout. At 1 h following nocodazole wash out, cells were fixed and stained to reveal MT networks. At steady-state, MT networks appeared similar when examining control *versus* fibroblasts from ARSACS patients (Fig. 4*A*). However, at 1 h following nocodazole treatment, differences were observed in the organization of MTs (Fig. 4*A*). MTs in ARSACS fibroblasts were disorganized, clustered, and nonradial, compared to the radial and regular network of MTs in control fibroblasts.

Since we observed altered MT organization in ARSACS fibroblast, we tested if sacsin has the ability to bind MTs and/or to regulate MT dynamics. Given the large size of the sacsin protein, we adapted a classical method of isolating MT-associated proteins (MAPs). Starting from brain lysates, cycles of temperature-dependent polymerization and depolymerization of MTs in the presence of GTP and glycerol, coupled to high speed centrifugation, significantly enriches MAPs (20). Using this method, we enriched sacsin and MAP1A (Fig. 4*B*). We also performed affinity-binding assays by incubating highly purified, Taxol-stabilized MTs with soluble rat brain lysate, followed by pelleting MTs and their MAPs by centrifugation. Using this approach, we could readily cosediment sacsin with MTs, with GAPDH as a control for a nonsedimenting protein (Fig. 4*C*).

Since sacsin interacts with MTs, we tested to see if sacsin regulates MT dynamics. MT end-binding protein 3 (EB3) caps the plus ends of growing MTs. Monitoring the dynamics of fluorescently labeled EB3 is routinely used to measure MT dynamics (21). EB3-GFP was transfected into WT and sacsin KO HeLa cells, and live cell imaging was performed to quantify the growth rate of MTs (Fig. 4, *D* and *E*). The TrackMate plugin of FIJI software was used to plot the tracks of EB3-GFP as indicated in Figure 4*D*. By applying a robust MT tracking protocol, we observed that the growth rate of MTs in sacsin KO cells was slower than WT cells (Fig. 4*E*).

**Supplemental Figure S5:**
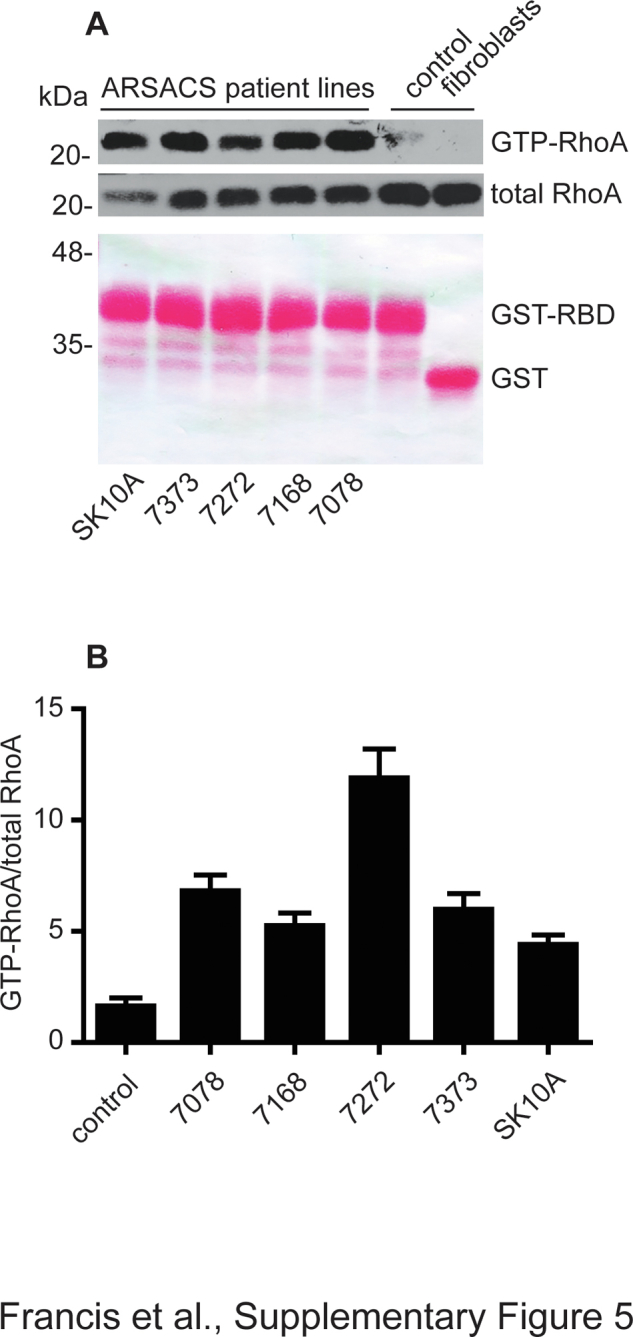
**Affinity pull down of active Rho-GTP.***A*, lysates prepared from ARSACS patient and control fibroblast were incubated with previously purified GST-Rhotekin conjugated to GST beads. Pull down is a representative of three experiments. Blots were then probed with RhoA. Ponceau S staining reveals the levels of GST-Rhotekin. *B*, quantification representing the ratio of GTP-Rho to total Rho.

The Rho family of GTPases are known to play an important role in the regulation of MT assembly status. For example, MT disassembly, such as that induced by nocodazole treatment, is known to lead to enhanced activation (GTP-bound form) of Rho (22). There are numerous effectors of Rho including ROCK-I and ROCK-II that bind Rho selectively in the active, GTP-bound form (23). The Rho-binding domain of ROCK-1 expressed as a GST fusion protein (RBD-GST) was used to pull down GTP-Rho in control and ARSACS fibroblasts. Interestingly, ARSACS patient fibroblasts have relatively high levels of GTP-Rho compared to control fibroblasts (Fig. S5, *A* and *B*). These results suggest that sacsin binds to MTs and regulates their dynamics, which in turn controls organelle positioning.

### Sacsin is required for lysosome tubulation

**Figure 5:**
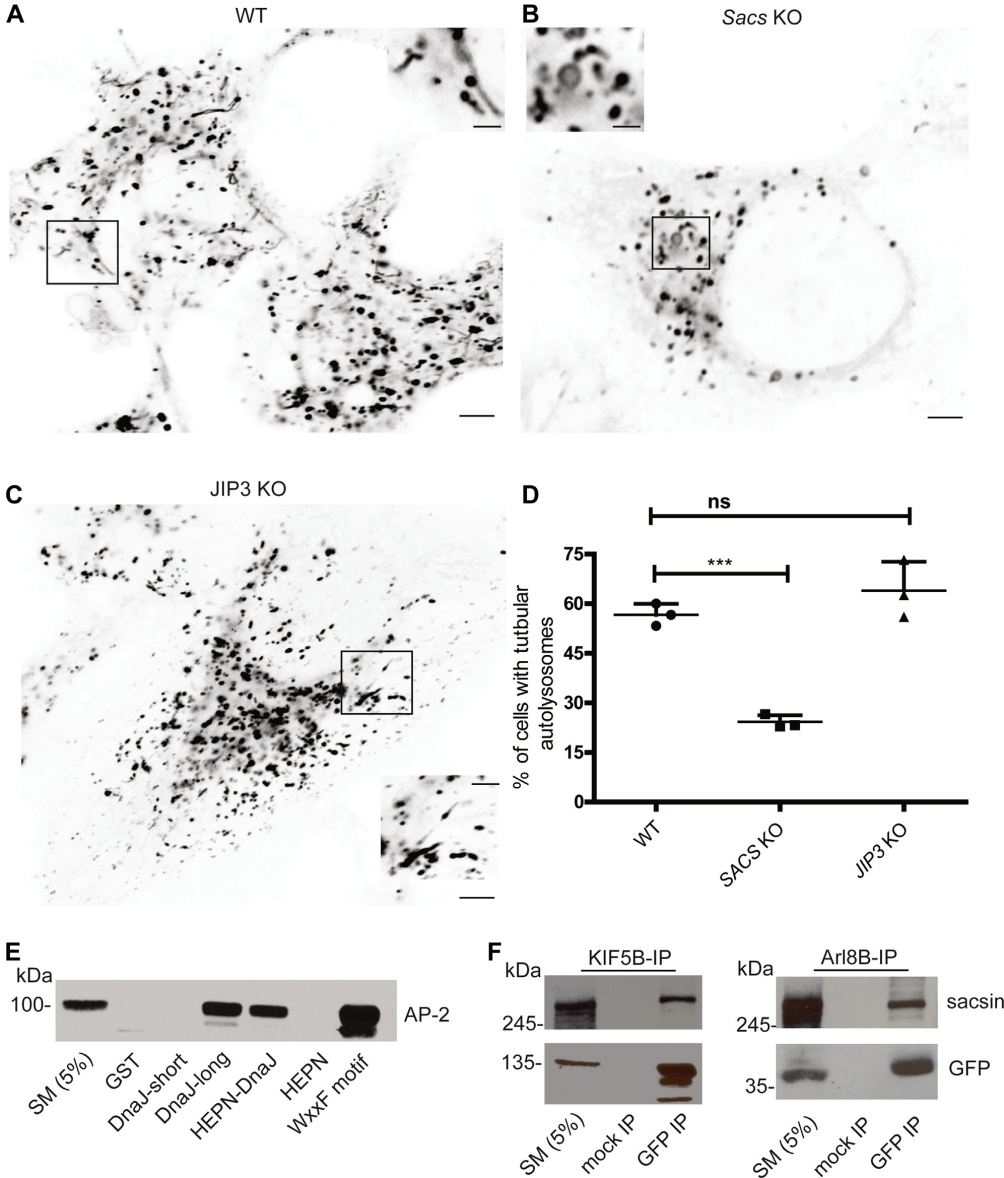
**Autolysosome reformation is deficient in sacsin KO cells.***A*, representative image from time lapse microscopy of lysotracker-568 staining in WT HeLa cells starved for 8 h. *B*, representative image from time lapse microscopy of lysotracker-568 staining in sacsin KO cells starved for 8 h. *C*, representative image from time lapse microscopy of lysotracker-568 staining in JIP3 KO cells which were starved for 8 h. The scale bar represents 10 μM and 3 μM for low- and high-magnification images. *D*, quantification showing the number of ALR tubules in WT, sacsin KO, and JIP3 KO cells. n > 40 cells from three independent experiments were quantified. Error bars indicate the SD ∗∗∗*p* < 0.0001. *E*, pull down showing the interaction of sacsin with AP2. *F*, lysates prepared from HEK-293 cells transfected with Kinesin-5B-GFP and ARL-8-GFP were processed for immunoprecipitation with antibody recognizing GFP. The precipitated samples were processed for immunoblot with antibodies recognizing sacsin and GFP as indicated. Immunoprecipitation shows the interaction of sacsin with KIF5B and ARL8.

**Supplemental Figure S6:**
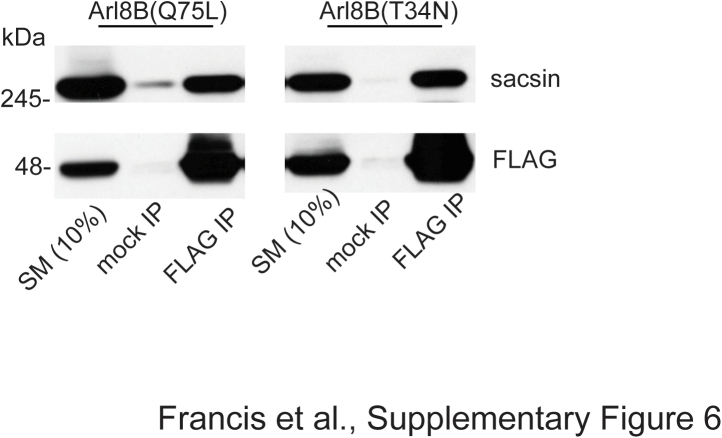
**Lysates prepared from HEK-293 cells transfected with FLAG-ARL8 (Q75L) or FLAG-ARL8(T34N) were processed for immunoprecipitation with antibody recognizing GFP.** The precipitated samples were processed for immunoblot with antibodies recognizing Flag and sacsin as indicated. An aliquot of the cell lysates (starting material, SM) equal to 10% of that added to the immunoprecipitates were analyzed in parallel. The apparent molecular mass of sacsin and the Flag-tagged constructs are indicated.

**Supplemental Figure S7:**
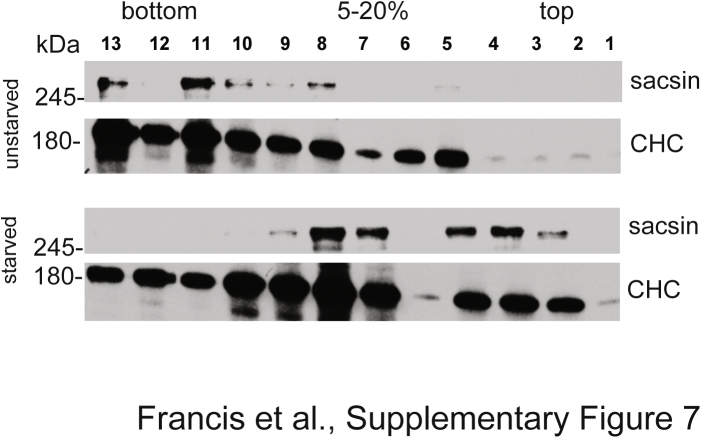
**Density gradient fractionation of 293-T.** Lysates were prepared from HEK-293T cell under basal and starvation conditions and loaded on top of a 5 to 20% sucrose gradient. Fractions were collected, resolved by SDS-PAGE, and processed for Western blotting with sacsin and CHC antibodies.

Following fusion of autophagosomes with lysosomes, the last step in autophagy, lysosome homeostasis is maintained through a process termed autolysosome reformation (ALR), which involves the formation of nascent lysosomes from pre-existing lysosomes (24). The first step in ALR involves the formation of tubules from autolysosomes, which subsequently bud off into small protolysosomes. Protolysosomes contain autolysosome components that mature and acquire acidity to form fully functional lysosomes. ALR is a recently discovered cellular process and the mechanistic details are not clearly understood. Spinster, mTOR, clathrin, and the clathrin adapter AP2 have been shown to mediate ALR in a MT-dependent manner (24). Since MT dynamics are altered in sacsin KO cells, loss of sacsin could also affect ALR. To investigate this hypothesis, ALR was initiated in sacsin KO and WT cells through nutrient deprivation (25). Loss of sacsin reduces formation of tubules budding from lysosomes (Fig. 5, *A*, *B* and *D*; Videos S6 and S7). Loss of LAMP1 tubules in sacsin KO cells are similar to cells treated with nocodazole (Video S9). Previous reports have demonstrated that clathrin and AP2 are important for ALR formation (26). In a mass spectrometry screen for binding partners of the DNAJ-HEPN domain of sacsin, we identified AP2 as an interacting partner. We confirmed the interaction using various GST-DNAJ-HEPN fusion proteins and mapped the AP2-binding site to a region between the DNAJ and HEPN domain (Fig. 5*E*). Kinesin motor protein (KIF5B) and Arl8 are important for ALR initiation. To further test the role of sacsin in ALR, we performed immunoprecipitation to test if it interacts with these proteins (27). Immunoprecipitation reveals interaction of sacsin with KIF5B and ARL8 (Fig. 5*F*). Since ARL8 cycles between active and inactive forms, we tested to see if interaction with sacsin is nucleotide dependent (28). Immunoprecipitation reveals ARL8 interaction with sacsin is nucleotide independent (Fig. S6). We also performed density gradient fractionation of 293T cell lysates under basal and ALR inducing starvation conditions and immunoblotted for clathrin and sacsin proteins. We observed greater cofractionation of sacsin with clathrin under starvation conditions (Fig. S7). We also tested to see if JIP3, which functions in MT dynamics, also regulates ALR. We initiated ALR with nutrient deprivation in JIP3 KO cells and observed no deficits in ALR (Fig. 5, *C* and *D* and Video S8).

## Discussion

Since the initial discovery of ARSACS in the Charlevoix and Saguenay regions of Quebec, patients have been reported worldwide and over 200 mutations have been identified, which span the entire protein. Analysis of the sacsin protein domain structure suggests that sacsin may function as a molecular chaperone and regulate protein quality control mechanisms in the cell (29). However, the cellular phenotypes seen in cells with disrupted sacsin function, which include accumulation of hyperfused mitochondria, nonphosphorylated neurofilament bundling in the soma, mispositioning of nuclei, Golgi fragmentation, and vimentin bundling suggest a transport/cytoskeletal function for sacsin (9, 10, 11). Vimentin associates with mitochondria and participates in the association of mitochondria with MTs, and the loss of vimentin causes mitochondrial fragmentation (30).

To test the hypothesis that sacsin could be involved in organelle transport, we generated sacsin KO cell lines and characterized the cellular distribution of organelles in the cells. Our observation of lysosome mispositioning and reduced motility upon loss of sacsin support the transport function hypothesis. MTs regulate the motility of organelles (31). Retrograde and anterograde MT motors have differential specificity toward the various posttranslational modifications of MTs. For example, lysosomes are selectively enriched over a subset of detyrosinated MTs, facilitating the fusion of autophagosome with lysosomes (32). The lysosomal mispositioning phenotype in HeLa cells observed here is robust, whereas in neurons, the major alteration is decreased lysosome motility and not overall repositioning. This difference could be attributed to the presence of different subsets of posttranslationally modified MTs as HeLa cells lack detyrosinated MTs (33). Moreover, it has been reported that p62 levels are decreased with enhanced autophagic flux in ARSACS fibroblasts (11, 34). MTs, which are linear polymers of α and β tubulin heterodimers are known to regulate a wide variety of cellular functions including organelle transport, cell polarity, motility, and chromosome segregation. MT dynamics are regulated by the intrinsic GTPase activity of tubulin as well by associated MAPs (31). We demonstrate that sacsin binds to MTs and regulates their dynamics. Given the large size of sacsin and the presence of multiple internal ATPase domains, the ability of the protein to interact with a number of organelle adapters including kinesin and Arl8 support our hypothesis. Zebrafish and *Caenorhabditis elegans* models have revealed increased axonal lysosomal abundance upon loss of JIP3. JIP3 plays a role in promoting amyloid plaque morphology (18). JIP3 interaction with p150^glued^ dynein subunit, and dynein intermediate light chain, suggest a retrograde transport function for JIP3 (16, 35, 36). Interaction of sacsin with JIP3 and similar defects in retrograde transport of lysosomes upon loss of sacsin suggest that although the primary function of sacsin might be to regulate MT dynamics, sacsin–JIP3 interaction is also important for lysosome specific transport.

Having identified a role for sacsin in MT dynamics, we tested to see if ALR, which is a MT-dependent processes, is also affected upon loss of sacsin. We believe that through its interaction with AP2, sacsin helps recruit the machinery required for ALR and at the same time, by modulating MT dynamics, generates the necessary force to tubulate lysosomes. The ALR phenotype seen in sacsin KO cells is very similar to that seen in cells upon nocodazole treatment, which completely depolymerizes MTs. A number of neurodegenerative diseases such as Parkinson’s and Alzheimer’s disease are characterized by deficits in axonal transport related to factors that reduce MT stability. Our results suggest that MT stabilizing agents may improve axonal transport as a disease therapy for ARSACS.

## Experimental procedures

### Cell lines and mouse strains

HEK-293 and HeLa cells were from ATCC (CRL-1573 and CCL-2). ARSACS patient fibroblasts were provided by J Paul Chapple (Queen Mary University of London). Sacsin KO mice were previously described (10). E18 rats were acquired from The Jackson Laboratory. All animal procedures were approved by the Animal Care Committee of the Montreal Neurological Institute at McGill University as per the Canadian Council on Animal Care.

### Antibodies and other materials

Sacsin mouse monoclonal antibody (WB-1:1000) was from Santa Cruz Biotechnology (sc-515118). JIP3 rabbit antibody (WB-1:300) was from Novus Biologicals (NBP1-00895). LC3 rabbit (IF-1:400) was purchased from Novus Biologicals. Mouse LAMP1 antibody (IF-1:500) was purchased from Abcam (ab25630). Anti-TOM20 (IF-1:1000) was purchased from Abcam (ab56783). ABCD3 antibody (IF-1:1000) was purchased from Abcam (ab211533). Mouse endosomal antigen 1 (EEA1) (IF-1:1000) was purchased from Abcam (ab2900). Mouse monoclonal Flag (M2) (IP- 0.2 μg/ml; WB- 1:10,000) was purchased from Sigma (F3165). Mouse Anti-Tubulin antibody (IF: 1:1000) was purchased from Sigma (T9026). Rat monoclonal anti-Hsc70 was purchased from Enzo (ADI-SPA-815-F). Clathrin heavy chain antibody (WB-1:1000) was purchased from Cell Signaling Technology (#2410S). Anti RhoA antibody (WB: 1:2000) was purchased from Cell signaling Technology (#2117). Rabbit polyclonal GFP (IP- 0.2 μg/ml; WB-1:10,000) was purchased from Invitrogen (A-11122). Plasmids from Addgene: pcdna3 Flag Jip1b (Addgene 52123), pcdna3 Flag Jip2 (Addgene 52123), pcdna3 Flag Jip3b (Addgene 53458), pDEST47-ARL8A-GFP(Addgene 67403), and dsEGFP-EB3-7 (Addgene 56474). Flag-Jip4 was generated by amplifying the Jip4 ORF from pCDNA3-T7-Jip4 (Addgene 58925) and cloning it into BamH1 and EcoRI sites of pCMV-Tag 2A. FLAG-ARL8 (Q75L) or FLAG-ARL8 (T34N) constructs were generated by site-directed mutagenesis. Positive clones were verified by Sanger sequencing. LysoSensor Green DND-189 (L7535) was purchased from Invitrogen.

### Cell culture and transfection

HeLa cells were cultured in Dulbecco’s modified Eagle’s medium with 10% calf serum, 50 units per ml penicillin, and 50 μg per ml streptomycin. Embryonic day 18 mice/rat neuronal primary cultures were prepared and maintained in neurobasal medium supplemented with B-27, N-2, L-glutamine, and antibiotic-antimycotic (Thermo Fisher Scientific). Disassociated single cell suspensions were plated on 35 mm glass bottom dishes previously coated with poly-L-lysine. Neurons were maintained regularly by replacing one half of medium with fresh neurobasal medium with supplements. Routine transfections were performed using Lipofectamine 2000 reagent (Invitrogen) as per manufacturer guidelines, and cells were analyzed 24 to 48 h post transfection.

### Production and use of lentivirus

Lentivirus-mediated KD of genes was performed as described previously. shRNAmiR sequences were designed based on the algorithm from Invitrogen and were cloned into pcDNA6.2/GW-emGFP-miR cassette. The emGFP-miR cassette was then subcloned into pRRLsinPPT lentiviral expression vector. The following sequences were used for shRNAmiR: nontargeting control AATTCTCCGAACGTGTCACGT, JIP3shRNA1 GTTTTGGCCACTGACTGAC, and JIP3 shRNA2 GTTTTGGCCACTGACTGA. The following packaging plasmids were used for lentivirus production in HEK-293T cells: pMD2.G, pRSV-Rev, pMDLg/pRRE. Medium containing virus particles was filtered using 0.45-μm filter and ultracentrifuged to concentrate the virus. Virus titer was calculated, and MOI5 was used for transduction of epithelial cell lines and MOI1 was used for transduction of rat primary neurons. For transduction of neurons, media was supplemented with polybrene 5 μg/ml. All experiments were subsequently carried out 5 days post transduction. Validation of knock down was confirmed by Western blot and qPCR.

### Generation of sacsin/JIP3 KO lines

PX459 V2.0 plasmid was obtained from Addgene (62988). The following gRNA primer 5′ – CACCGAGAAGTGATCATGGAGACCA-3′ was cloned into PX459 using BbsI overhangs. HeLa cells were transfected with the plasmid followed by puromycin selection (1.5 μg/ml) 48 h post transfection. Cells were then seeded into 96-well plates to ideally obtain one cell per well, which were then subsequently expanded and screened for KO clones. For generation of JIP3 KO line, three synthetic gRNAs (Synthego) were used: GACTTGGTGCCTGTGGTGCT, GGCTGCAGGCTCTCGTTGAG, and TGACCTGTACTTCCATGTTG. To prepare the RNP complexes, Cas9 2NLS nuclease (*Streptococcus pyogenes*) (Synthego) and the reconstituted sgRNA were added to the Nucleofector solution (Lonza). Mixture was incubated at RT for 15 min and added to freshly harvested HeLa cells and electroporated using 4D X Unit (Lonza). Positive KO clones were screened by PCR using primer flanking the cut sites.

### Immunofluorescence microscopy

Cells were grown on poly-L-lysine (PLL)/fibronectin-coated coverslips, washed with PBS, and fixed using warmed 4% paraformaldehyde. Cells were then permeabilized with 0.1% Triton-X-100 and blocked with 2% bovine serum albumin for 1 h at RT. Fixed cells were stained with primary antibody at 4 °C overnight and then stained with secondary antibody for 1 h at room temperature. Cover slips were then washed and mounted on slides. Images were then acquired on a Zeiss LSM 880, Axio Observer microscope or Leica confocal microscope. WT and sacsin KO mice brains were removed after perfusion fixation with 4% paraformaldehyde. Fixed brains were then embedded and sectioned; immunohistochemistry was performed using anti-LC3 antibody overnight followed by staining with a secondary antibody and processed for imaging.

### Coimmunoprecipitation

Transfected HEK-293T cells were lysed in lysis buffer (20 mM Hepes, 150 mM NaCl, 1 mM DTT, and 1% Triton-X-100, supplemented with protease and phosphatase inhibitors, 5 mM sodium pyrophosphate, 0.5 μM okadoic acid, 1 mM Na_3_VO_4_, and 10 mM NaF). Cell lysates were then clarified by centrifugation at 234,000*g* for 15 min. Supernatants were then incubated with 10 μl Protein A/G Sepharose (GE Healthcare) and 5 μg of anti GFP polyclonal or FLAG (M2) monoclonal antibody. Beads were washed, and proteins were resolved by SDS-PAGE and processed for autoradiography.

### Electrophoresis and immunoblotting

Cells were pelleted and washed with ice cold PBS, resuspended, and lysed in lysis buffer (25 mM Tris–HCl, pH 7.5, 150 mM NaCl, 1% Triton X-100 (wt/vol) with protease inhibitors). After incubating the cells with lysis buffer for 15 min at 4 °C, cell lysate was centrifuged at 21,000*g* for 15 min and supernatant was resolved by SDS-PAGE and analyzed by Western blotting.

### Quantifying lysosomal distribution

For estimating the lysosome spatial distribution from the center of the cell to periphery, cumulative lysosome channel intensity was estimated in 10% increments from the center and measured. In order to achieve this, first the nucleus and edge pixels that define the boundary of the cell was defined. Images were then preprocessed in order to reliably obtain the cell boundary. Preprocessing was done in the following order.

#### Erosion

To remove the connected cell processes extending from a cell to another hindering the segmentation of the cell.

#### Clustering

After applying the erosion, a connectivity-based clustering is performed. Connectivity-based clustering is a simple method where connected pixels are grouped in the same cluster and an ID is provided to the cluster. The clusters are sorted based on the size, holes in the clusters are patched, and small clusters are removed from the image. Then, we iterate over all the cluster in the image.

#### Contour approximation

For each cell cluster, we use the opencv implementation of Douglas-Peucker algorithm to approximate the periphery of the cell. The closeness of the approximated contour to the original cell boundary can be controlled using the parameter. We identified that value of 0.009 is enough for capturing the cell periphery and reduces that number of points to be required for defining the contour.

Identifying the cell center—after the contour approximation, the user is presented with the original image with the cell and prompted to identify the cell center by clicking at the center of the cell. Then, a vector method for distance estimation is used to identify points with 10% increments from the cell center to the periphery. (Distance from center to periphery).

$${\text{p}}_{\text{inc}}=\text{ d∗}\left({\text{p}}_{\text{center}}-{\text{p}}_{\text{periphery}}^{\left(\text{i}\right)}\right)$$

$${\text{p}}_{\text{new}}{=\text{p}}_{\text{center}}{+\text{p}}_{\text{inc}}$$

Where p_inc_ - a increment along vector from cell center (p_center_) to a point on the periphery (${\text{p}}_{\text{periphery}}^{\left(\text{i}\right)}$), d - distance increment, and p_new_ is the new point.

Intensities contained in each increment is estimated and normalized such that the intensities are in the range of 0 to 1.

### Mass spectrometry analysis

To identify interacting partners of sacsin, immunoprecipitation was performed using affinity-purified sacsin antibody followed by mass spectrometry analysis. Briefly, mouse brain lysates in lysis buffer were incubated with Protein A Sepharose beads conjugated to sacsin antibody for 2 h at 4 °C. Beads were then washed in lysis buffer, resuspended in Laemmli sample buffer, and submitted to mass spectrometry analysis at the McGill University Health Centre. As a control, Protein A Sepharose beads were incubated with lysates. Identified sacsin-interacting partners are provided in Supplementary Data File 1. RAW data are available *via* ProteomeXchange consortium with identifier PXD033823.

### LysoSensor DND-189 assay

LysoSensor DND-189 was purchased from ImmunoChemistry Technologies, LLC. Cells were plated the previous day and were then incubated with LysoSensor DND-189 and visualized by fluorescence microscopy.

### Neuron live cell imaging

For primary neuronal cultures, E18 embryos from WT and sacsin KO mice were used. Brains were dissected and dissociated into single cell suspension, neurons were plated at a density of 300,000/well on PLL-coated 35-mm No. 1.5 glass-bottom dishes (MatTek Corporation) and grown in Neurobasal medium supplemented with B-27, N-2, L-glutamine (500 μM), and penicillin/streptomycin. For live cell imaging of lysosome, neurons were labeled with 1 μM lysotracker for 1 h at 37 °C and was then washed with imaging buffer 10 mM Hepes, 10 mM glucose, 1.2 mM CaCl_2_, 3 mM KCl, 1.2 mM MgCl_2_, 145 mM NaCl previously adjusted to pH-7.4. Imaging was done with the same buffer to reduce background fluorescence. Time lapse images were generated as 15 frames/s video files. Images were processed and Kymographs were generated by ImageJ (NIH).

### Kymograph and track quantification

Kymographs were generated from live cell images of DIV8 neurons using ImageJ, and segmented lines were drawn along 25 μm region from the distal part of the AIS. Axons and dendrites were determined based on length with axons being determined as three times longer than dendrites. Anterograde movements were oriented from left to right. Number of events for anterograde and retrograde lysosomal movement in axons and dendrites were quantified from the kymographs as described earlier from several cells (37). Images were analyzed from three independent experiments.

### Expression analysis

Total RNA was extracted from cells using RNeasy Mini kit (Qiagen), and 1 μg of RNA was reverse transcribed using iScript Reverse Transcription Supermix (Bio-Rad Laboratories). SsoFast EvaGreen Supermix (Bio-Rad laboratories) was used to amplify the DNA on Bio-Rad CFX Connect Real-Time PCR Detection System. Samples were run in triplicates and expression was normalized relative to control cells using endogenous gene GAPDH. The following primers were used: JIP3-F, 5′-ACAGGATGAAATGTCCGAGTCAGG-3′ and JIP3-R, 5′- GGTGACCTGTACTTCCATGTTG-3′ respectively.

### Quantification of MT growth rates in EB3-GFP-transfected cells

HeLa cells were transfected 48 h before imaging with 2 μg of plasmid DNA for EB3-GFP. Images were obtained using the fast airyscan of the inverted Zeiss LSM 880 microscope (Carl Zeiss Inc) with a 37 °C incubation chamber and a 63×/1.4 NA objective. 488-nm lasers were used to acquire the images at 1 frame/s for 5 min. MT growth rates were measured on time lapse movies of EB3 in HeLa cells. The EB3-GFP comets at MT plus-ends were tracked using TrackMate plugin of FIJI software according to the detailed MT tracking protocol established elsewhere (https://escholarship.mcgill.ca/concern/theses/dn39x375m). Shortly, the images were preprocessed using the difference of Gaussians filter to remove the background noise. The EB3 comets were tracked using Trackmate plugin. The tracking measurements for MT dynamics such as track duration, displacement, and speed were extracted and saved in excel sheet. Statistical analysis was performed using an R script that takes this data and calculates the growth rates.

### MT binding assay

To determine if sacsin binds to MTs, we adopted the method according to Neely and Boekelheide to purify MAPs from rat brain (38). Briefly, MTs and MAPS were polymerized by repeated cycles of temperature-dependent assembly and disassembly in the presence of Taxol, glycerol, and GTP following centrifugation through sucrose. To analyze the interaction between sacsin and MTs, MT cosedimentation assay was performed using preformed Taxol-stabilized MTs (Cytoskeleton Inc). High *g* spun (130,000*g*) rat brain lysate was incubated with Taxol-stabilized MTs and then centrifuged at 100,000*g* for 15 min at 25 °C. Supernatant and pellet fraction were resolved by SDS-PAGE and analyzed by Western blotting using anti-sacsin and MAP1A antibodies.

### MT polymerization and regrowth in cell lines

Patient-derived fibroblast were grown on PLL-coated coverslips and treated with 20 μM nocodazole for 2 h to depolymerize the MTs and fixed for immunofluorescence using anti-tubulin antibody. For MT regrowth assay, patient fibroblasts were first incubated with nocodazole and then washed to remove nocodazole followed by fixation after 1 h and processed for immunofluorescence with anti-tubulin antibody.

### Statistical analysis

Data processing was performed using Excel and GraphPad Prism (GraphPad software). *t* test and one-way ANOVA test were performed for statistical analysis.

## Data availability

All data are contained within the article.

## Supporting information

This article contains supporting information.

## Conflict of interest

P. S. M. is a distinguished James McGill Professor and Fellow of the Royal Society of Canada. The authors declare that they have no conflict of interest.

## Supporting information

Video S1 Video showing LysoTracker dynamics in WT neurons. Images were acquired at 60 frames/min and played at 5 frames/s. Bar, 15 μm.

Video S2 Video showing LysoTracker dynamics in SACS KO neurons. Images were acquired at 60 frames/min and played at 5 frames/s. Bar, 15 μm.

Video S3 Video showing LysoTracker dynamics in SACS KD neurons. Images were acquired at 60 frames/min and played at 5 frames/s. Bar, 10 μm.

Video S4 Video showing LysoTracker dynamics in control shRNA-miR–transduced neurons. Images were acquired at 60 frames/min and played at 5 frames/s. Bar, 10 μm.

Video S5 Video showing LysoTracker dynamics in JIP3 targeting shRNA-miR KD neurons. Images were acquired at 60 frames/min and played at 5 frames/s. Bar, 10 μm.

Video S6 Video showing autolysosome reformation in WT HeLa cells starved for 8 h. Lysosomes were labeled with lysotracker. Images were acquired at 60 frames/min and played at 25 frames/s. Bar, 10 μm.

Video S7 Video showing autolysosome reformation in sacsin KO cells starved for 8 h. Lysosomes were labeled with lysotracker. Images were acquired at 60 frames/min and played at 25 frames/s. Bar, 10 μm.

Video S8 Video showing autolysosome reformation in JIP3 KO cells starved for 8 h. Lysosomes were labeled with lysotracker. Images were acquired at 60 frames/min and played at 25 frames/s. Bar, 10 μm.

Video S9 Video showing autolysosome reformation in WT HeLa cells starved for 8 h in presence of 20 μM nocodazole. Lysosomes were labeled with lysotracker. Images were acquired at 60 frames/min and played at 25 frames/s. Bar, 10 μm.
