# Correction: The ARSACS disease protein sacsin controls lysosomal positioning and reformation by regulating microtubule dynamics

**DOI:** 10.1016/j.jbc.2022.102718

**Published:** 2022-11-28

**Authors:** Vincent Francis, Walaa Alshafie, Rahul Kumar, Martine Girard, Bernard Brais, Peter S. McPherson

The author list should read as follows:

Vincent Francis^1^, Walaa Alshafie^1^, Rahul Kumar^1^, Martine Girard^1^, Eric Krochmalnek^1^, Bernard Brais^1^, Peter S. McPherson^2^

The author contributions should read as follows:

*Author contributions* - V. F., B. B., and P. S. M. conceptualization; V. F. and P. S. M. methodology; V. F. and P. S. M. writing–original draft; V. F., W. A., R. K., and M. G. visualization; V. F., W. A., R. K., M. G. and E.K. investigation; V. F., W. A., R. K., and M. G. validation; B. B. and P. S. M. supervision; V. F. and P. S. M. writing–review and editing; R. K. software; V. F. and P. S. M. data curation.

